# Perceptions of the health risk from hot days and the cooling effect of urban green spaces: a case study in Xi’an, China

**DOI:** 10.3389/fpubh.2023.1211164

**Published:** 2023-08-22

**Authors:** Tian Zhang, Rong Huang, Mei Yang, Guohua Lin, Xiaoyan Ma, Xuan Wang, Qian Huang

**Affiliations:** ^1^Northwest Land and Resource Research Center, Shaanxi Normal University, Xi’an, China; ^2^School of Geography and Tourism, Shaanxi Normal University, Xi’an, China

**Keywords:** hot days, urban green spaces, health risk perception, cooling effect, Xi’an City

## Abstract

**Background:**

Hot days are one of the typical threats to human health and sustainable cities. The exploration of residents’ perceptions of thermal environment and its mitigation measures will support the health risk prevention.

**Methods:**

A survey with a combination of closed-ended and open-ended questions was conducted in July 2021 among 13 urban parks in Xi’an City, China. With the help of ANOVA and ordinal logistic regression, this study investigated the influencing factors both on residents’ health risk perception of hot days and their perception of the effect of urban ecological landscape on reducing the thermal risk. The relationship between health risk perception and residents’ needs of urban ecological construction was also explored.

**Results:**

According to 325 valid questionnaires, the male-female ratio of respondents was found to be 1:0.87, young people aged 18-29 (26.46%), the retirees (27.08%) and the ones with undergraduate education (33.23%) were, relatively, the largest groups. The results show that 92.31% of the respondents believed that their daily lives were under the influence of hot days. Housing types, occupation, cooling equipment at work, and outdoor working hours all had a significant impact on their high temperature perceptions. The proportion of respondents who were under a huge health risk and sought medical treatment due to hot days was 30.16% and 44.92%, respectively. Women were 18.52 and 2.33 times more likely to suffer health threats and experience discomforts than men. Furthermore, 73.23% of the respondents believed that the urban ecological landscapes in Xi’an had an enhanced cooling effect in recent years. Compared with the morphological characteristics, residents’ recognition of the restriction of landscape’s area on its cooling effect was higher, and the residence duration showed a significant influence.

**Conclusion:**

The cooling effect of green spaces and water effectively resisted urban thermal threats, and residents’ needs of the urban ecological landscapes was associated with their health risk perceptions of hot days. In the future, it is necessary to promote the early warning of hot days, meanwhile, the optimization of landscape patterns of green infrastructures should be implemented in urban planning for the purposes of residents’ health risk prevention.

## 1. Introduction

Against the background of global warming and accelerated urbanization, the increase in urban temperature has significantly exceeded that of the global temperature (1). According to the Sixth Assessment Report of the Intergovernmental Panel on Climate Change (IPCC), the global surface temperature during 2011–2020 was 1.09°C warmer than that during 1850–1900. As one of the most extreme meteorological events, hot day is an atmospheric process that exerted adverse impacts on human health, urban safety, and the ecological environment (2, 3). In addition, the combination of urbanization and global warming will aggravate the severity of hot days (4). “Good health and well-being” and “Sustainable cities and communities” are the important parts of Sustainable Development Goals (SDGs) of 2030. In recent years, hot days are showing a trend of increasing frequency, intensity, and duration (5), which have seriously hindered the achievement of the SDGs (6). At the same time, ecological landscape construction is considered to be an important natural-based solution (NbS) to urban thermal environmental stress. The cooling effect of urban green spaces and water has been widely confirmed (7, 8), especially the beneficial effects of urban green spaces on the mental and physical health of residents (9). Conducting comprehensive researches on urban thermal risk solutions from the perspective of urban ecological construction and residents’ health risk perceptions is a feasible way to build sustainable cities and against public health threats.

Perception of hot days and thermal risk is an important part of public risk perception. Directing attention to the needs of urban residents under thermal environmental risks can provide a reference for the government to formulate reasonable adaptation policies and effective urban ecological construction. International research on hot days perception and adaptation began in the 1980s, and qualitative interviews with vulnerable groups have become the focus in recent years (10, 11). As Chinese society gradually attaches importance to improving the quality of human settlements, relevant studies have attracted more attention (12). The current research mainly focuses on the awareness of high temperature weather and its influencing factors (13, 14), the health risks of hot days (15, 16), the impact of high temperature on residents’ behavior (17, 18), thermal risk adaptation (19, 20), mental and physical health benefits from urban green spaces (21, 22), and the mechanism of urban ecological construction promoting public health (23, 24). Conducting questionnaire surveys for specific groups (25), using case-crossover analysis (26), and carrying out a time-series assessment with mortality or morbidity data and meteorological data (16) were the main ways to explore the residents’ health risk perceptions and their adaptations. However, there are still certain issues among the existing researches that deserve to be further explored. On the one hand, existing studies on urban thermal risk perceptions has not well integrated with its nature-based solutions, nor has it paid enough attention to the judgment and needs of residents regarding the cooling effect of urban green spaces, thus making it difficult to combine the research results with the improvement of comfort in human settlements. On the other hand, one of the goals of the studies on residents’ health risk perceptions should be guide urban ecological construction and create a better living environment. However, there is still a lack of attention on the public cognition of ecological landscape patterns toward the mitigation of hot days. As such, the research on the health risk perceptions from the perspective of landscape ecology needs to be strengthened.

Xi’an is one of the three international metropolises in China. In conjunction with the rapid urbanization, the buildings in the central area of Xi’an are too dense since urban construction is mostly attached to, and overlapped with, historical sites, which aggravates the impact of hot days and thus makes it a “furnace” city (27). Existing studies have shown that the death cases of high temperature in Xi’an increased by 54% in 2010 compared with the same period the previous year (28). Based on the data from 2013 to 2015 on mortality, cities located at middle latitudes like Xi’an was found to have a higher mortality increase under exposure to hot days (29). And an extremely high temperature of 41.80°C on July 24, 2017 in Xi’an has posed great challenges to public health (30). In view of this, against the demand of reducing the human health risks and the construction of sustainable cities, three detailed objectives were defined in this study: (1) to investigate residents’ perception of health risk caused by hot days and its influencing factors; (2) to examine residents’ perception of the cooling effect of urban green spaces and its influencing factors; (3) to explore how would the residents’ health risk perception impact on their needs of the construction of urban ecological landscapes. The results of this study could help to explore the feasible ways to alleviate the human health risk of urban extreme high temperatures, which could also provide a scientific reference for promoting the optimization of human settlement and urban ecological construction in Xi’an.

## 2. Materials and methods

### 2.1. Study area

Xi’an City is located in the middle of the Yellow River Basin, with the Qinling Mountains in the south and the Loess Plateau in the north. The geographical location makes it prone to the “foehn effect.” Xi’an has a semi-humid continental monsoon climate in the warm temperate zone, with an annual average temperature of 13.10–14.30°C, and an annual precipitation of 528.30–716.50 mm. High temperature is one of the meteorological disasters that occur in Xi’an. Based on the monitoring of national meteorological station in the city (Jinghe Station, located at 34°26’N, 108°58’E in the north of the Weiyang District), the high temperature station frequencies, i.e., ≥35°C, was found to be 139 times in 28 days, 106 times in 20 days, and 147 times in 34 days during 2019 to 2021, respectively. It must be noted that these results increased significantly in 2021. In addition, the most severe hot days in Xi’an also usually occur in July. During 2019 to 2021, the monthly extreme low and extreme high temperature in July ranged from 18–40°C, 17–38°C, and 19–39°C, respectively. During the investigation period of this study, the weather in Xi’an was mainly sunny with the highest temperature being noted as 39°C.

**Figure 1:**
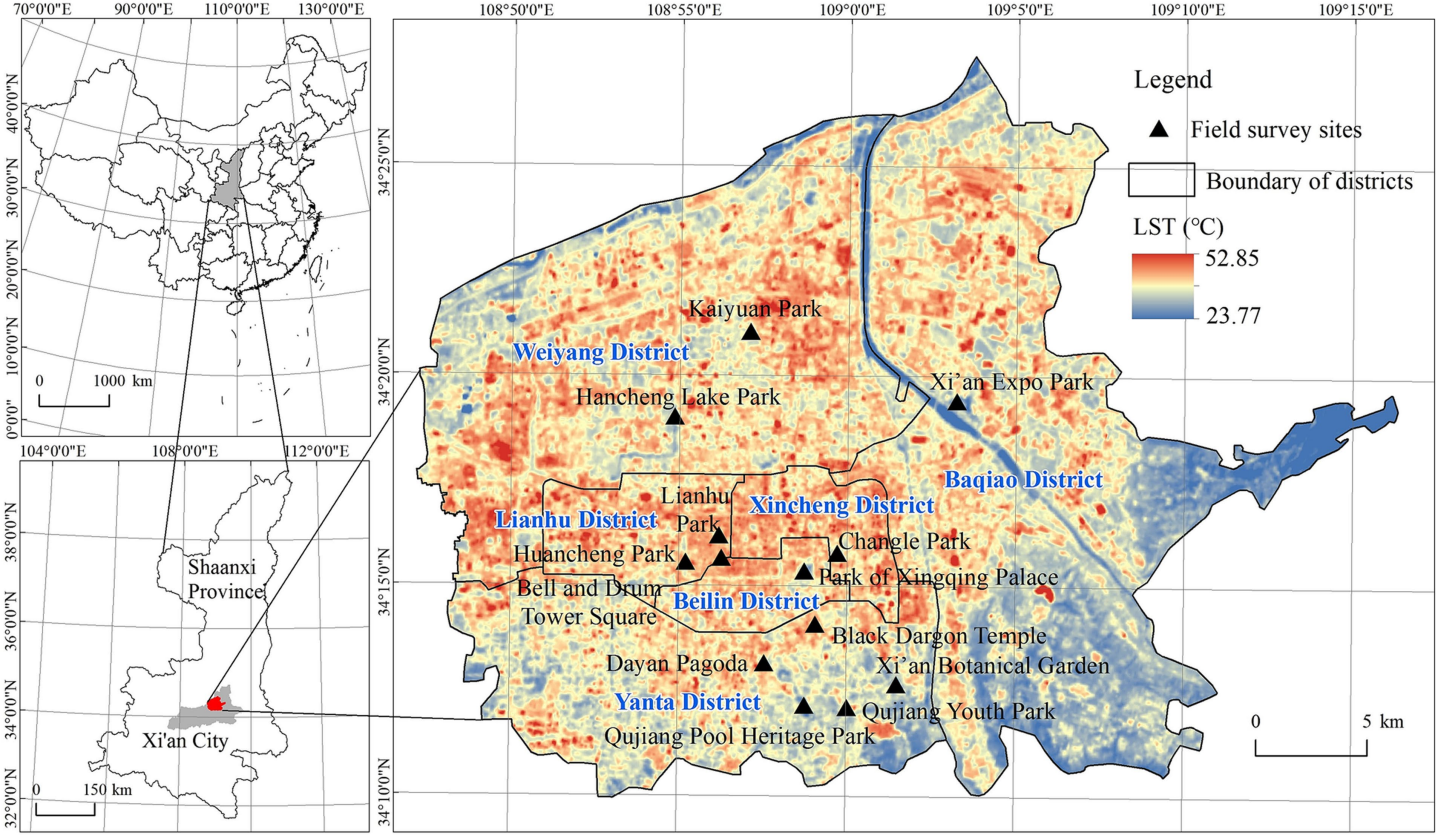
Study area and field survey sites.

In this study, the six districts (Weiyang District, Baqiao District, Lianhu District, Xincheng District, Beilin District, and Yanta District) were chosen as the study area. These districts are the core areas of population and of urban construction in Xi’an, with a total territory area of 844.50km^2^. They are also the place where hot days frequently occur and where the urban ecological parks are concentrated. Based on the cloud-free thermal infrared bands of Landsat 8 OLI/TIRS images, the land surface temperature (LST) was retrieved. Figure 1 shows that the LST ranged from 23.93 to 51.02°C and showed an average value of 33.41°C on 13 August 2019 in the study area (31). Low LST values were concentrated in urban water bodies and the undeveloped region in the east of Baqiao District. Based on a comprehensive consideration of location, area, passenger flow, the proportion of green spaces and water landscapes, and construction years, this study took the 13 typical urban parks in the six districts as examples. The selected samples all have high accessibility, high proportion of natural landscapes, and high popularity among residents, thus ensuring the cooling effect of the parks and sufficient respondents during the field survey. Then, field research on urban residents’ perceptions of hot days and the cooling effect of urban parks was carried out. The locations of the field survey sites are shown in Figure 1.

### 2.2. Questionnaire design and field survey

#### 2.2.1. Questionnaire design

In order to achieve the research objectives, this study designed a questionnaire of “Urban residents’ health risk perceptions of hot days and the cooling effect of urban green spaces and water landscape,” which includes 5 parts and 27 questions (see Supplementary material for the complete questionnaire). The closed-ended questions, open-ended questions and hybrid questions were set during the questionnaire design in this study. The answers of the closed-ended questions were divided into different levels or categories, and the respondents were invited to choose freely within the scope of questionnaires (e.g., the basic information, working and living conditions, and perceptions which needed to describe as degrees). Meanwhile, the open-ended questions focused on the specific evaluation and personal needs of the respondents (e.g., the temperature and duration of hot days, the scores of cooling effect, and the specific suggestions). And the hybrid questions were set to help respondents to supplement the answers that were not considered during the questionnaire design, which was set as the last item in a series of alternative answers in a form of filling in the blanks.

The main contents of the questionnaire included the following: (1) The basic information of the respondents (i.e., gender, age, physical condition, education, occupation, monthly income, and residence time in Xi’an); (2) the details of the respondents’ daily life and work (i.e., housing type, cooling equipment at home, working environment, cooling equipment at work, outdoor working hours, and commuting modes); (3) the health risk perception of hot days (i.e., duration of hot days, impact intensity of hot days, specific impact types of hot days, health threat of hot days, and specific types of physical discomfort caused by hot days); (4) the perception of the cooling effect of urban green spaces and water landscapes (i.e., the trend of cooling effect of urban green spaces and water landscapes, whether the area and the shape of landscape affects its cooling effect, as well as the difference of the cooling effect between urban green spaces and water); and (5) the respondents’ personal needs and suggestions (i.e., measures that residents want the government to take to deal with hot days, the necessity of building urban ecological landscapes, and other suggestions). Before formally launching the survey, we sent the questionnaire to 7 experts and scholars who have been engaged in the research of urban ecological environment for a long time, and improved the questionnaire according to their suggestions to ensure the rationality and legibility of the questionnaire.

#### 2.2.2. Field survey

In order to reflect the intuitive perceptions of urban residents on hot days, as well as the cooling effect of urban parks comprehensively, this study carried out a field survey in 13 typical urban parks in Xi’an during the period of 10:00–19:00 from July 26 to 31, 2021. During the research period, the weather was sunny, and the maximum daily temperature was mostly above 35°C. Furthermore, the questionnaires were handed out and completed face to face in this study. Respondents were randomly selected to fill out the questionnaires independently, and the investigators were responsible for the guidance. The investigators were trained before the survey that if the respondents were illiterate or unable to fill out the questionnaire independently, then the investigators would dictate the questions and fill in the answers on their behalf. The questionnaire would take about 5–10 min to fill out and which would then be reviewed after the on-site survey. The field survey was carried out during high temperature weathers in the six districts of Xi’an City. The survey objects were the residents in or around the urban parks. In addition, both the survey time and the respondents were representative. A total of 360 questionnaires were sent out in this survey, and 325 valid ones were collected, with an effective rate of 90.28%—excluding those with a rejection rate or omission rate of more than 20%. Around 25 questionnaires were completed in each urban park sample.

#### 2.2.3. Reliability test and validity test

Furthermore, SPSS 25.0 was used to do the reliability test and validity test of the scaled-response questions in the questionnaire. Specifically, the reliability of the data was tested by Cronbach’s α coefficient, and the closer it is to 1, the higher the reliability of the data (32). In this study, the Cronbach’s α coefficient of all the scaled-response questions was 0.82, and the Cronbach’s α coefficients of residents’ perception of hot days and the perception of ecological landscape’s cooling effect were 0.86 and 0.81, respectively, all of which are greater than 0.80, so the data of the questionnaires had a high reliability. In addition, this study used KMO value and Bartlett’s Test of Sphericity for the validity test. The KMO values of all the scaled-response questions, residents’ perception of hot days, and residents’ perception of ecological landscape’s cooling effect were 0.80, 0.84, and 0.78, respectively. And all the Bartlett’s sphericity test also passed the significance test (*p* < 0.001), indicating a good validity of the data, and further statistical analysis could be conducted.

### 2.3. Statistical methods

In this study, the quality control of the questionnaire results was achieved by a double recording method. Then, the SPSS 25.0 software was used for a statistical analysis on the questionnaire results. The methods used in the study included descriptive statistics, the analysis of variance (ANOVA), and logistic regression modeling. Additionally, *p* < 0.05 was regarded as the criterion of statistical significance. All the variables were coded from V1 to V22 and the specific variable assignments could be found in Table 1. The variable assignment rules were based on the classification or sequence characteristics of the answers set in the questionnaire. For instance, categorical variables were numbered according to the order in which they appeared in the questionnaire (e.g., 1 = male and 2 = female for gender), and the sequential variables were numbered from 1 according to the degrees they represented, from low to high (e.g., 1 = barely, 2 = minor, 3 = general, 4 = large, 5 = great for the health threat of hot days). Among the aforementioned, the descriptive statistics aimed to describe the basic characteristics of the respondents, including their basic information, as well as their living and working conditions in summer. ANOVA is the most widely used method of variance testing, the advantage of which is that it can simultaneously examine the significance of multiple indexes and also observe their interaction (33). In this study, the ANOVA was mainly used to judge whether there are statistical differences in the residents’ perceptions of hot days, as well as with respect to the cooling effect of urban green spaces and water landscapes among the residents with multiple characteristics. The variables coded from V1 to V13, and from V14 to V21 were taken as the independent variables and dependent variables during the difference analysis, respectively.

**Table 1:** Variables assignment for the ANOVA and logistic regression model.

Variables		Variable assignments
V1	Gender	1 = male, 2 = female
V2	Age	1 = <18, 2 = 18–29, 3 = 30–39, 4 = 40–49, 5 = 50–59, 6 = ≥60
V3	Physical condition	1 = great, 2 = well, 3 = general, 4 = have disease
V4	Education	1 = junior high school and below, 2 = high school, 3 = junior college, 4 = undergraduate, 5 = postgraduate and above
V5	Occupation	1 = public institutions, 2 = company employee, 3 = service industry, 4 = student, 5 = outdoor worker, 6 = retiree, 7 = individual business, 8 = unemployed, 9 = others
V6	Monthly income	1 = under 2000 CNY, 2 = 2000–4,000 CNY, 3 = 4,000–6,000 CNY, 4 = 6,000–8,000 CNY, 5 = over 8,000 CNY
V7	Residence time in Xi’an	1 = local people, 2 = within six months, 3 = six months to two years, 4 = three to five years, 5 = five to ten years, 6 = more than ten years
V8	District	1 = Yanta District, 2 = Beilin District, 3 = Lianhu District, 4 = Weiyang District, 5 = Baqiao District, 6 = Xincheng District
V9	Housing type	1 = non-top floor of building, 2 = top floor of building, 3 = flat building, 4 = villa, 5 = other
V10	Cooling equipment at home	1 = none, 2 = one kind, 3 = two kinds, 4 = three kinds
V11	Working environment	1 = outside in the sun, 2 = outdoor shade, 3 = indoor without air conditioner, 4 = indoor with air conditioner
V12	Cooling equipment at work	1 = none, 2 = one kind, 3 = two kinds, 4 = three kinds
V13	Outdoor working hours	1 = within 1 h, 2 = 1–2 h, 3 = 2–4 h, 4 = 4–6 h, 5 = 6–8 h, 6 = more than 8 h
V14	Influence intensity of hot days	1 = barely, 2 = minor, 3 = general, 4 = large, 5 = great
V15	Influence types of hot days	1 = no influence, 2 = single influence, 3 = multiple influences
V16	Health threat of hot days	1 = barely, 2 = minor, 3 = general, 4 = large, 5 = great
V17	Physical discomfort due to hot days	1 = asymptomatic, 2 = single symptom, 3 = multiple symptoms
V18	Trend of cooling effect	1 = getting better, 2 = not sure, 3 = getting worse
V19	Judgment of cooling ability	1 = green space has better cooling effect, 2 = unsureness, 3 = water has better cooling effect
V20	Landscape’s area affects cooling effect	1 = has impact, 2 = not sure, 3 = has no impact
V21	Landscape’s shape affects cooling effect	1 = has impact, 2 = not sure, 3 = has no impact
V22	Necessity of urban green space and water landscapes construction	1 = no need at all, 2 = not quite necessary, 3 = general, 4 = necessary, 5 = very necessary

The ordinal multi-category logistic regression model is a probabilistic nonlinear regression model, which is suitable for analyzing the relationship between an ordered multi-category dependent variable and multiple independent variables. The independent variable of the model can be continuous or discontinuous, and it is most suitable for the discrete and hierarchical dependent variable (34). In this study, the ordinal logistic regression models with multi-category dependent variables were built according to their continuous characteristics, among which the last category of each dependent variable and independent variable was set as the reference group. To detect the influencing factors of the urban residents’ health risk perceptions of hot days (V14-V17) and their cognitions of the cooling effect of urban ecological landscapes (V18, V20, and V21), the basic characteristics of the respondents involved in the survey were taken as the independent variables (V1-V13). It should be noted that V18 was not taken considered in a logistic regression due to the non-continuity. Moreover, the influence of residents’ cognition of thermal environment and its mitigation (V14-V21) on their needs of the urban green spaces and water construction (V22) was also identified by the logistic regression model.

## 3. Results

### 3.1. Basic characteristics of the respondents

**Figure 2:**
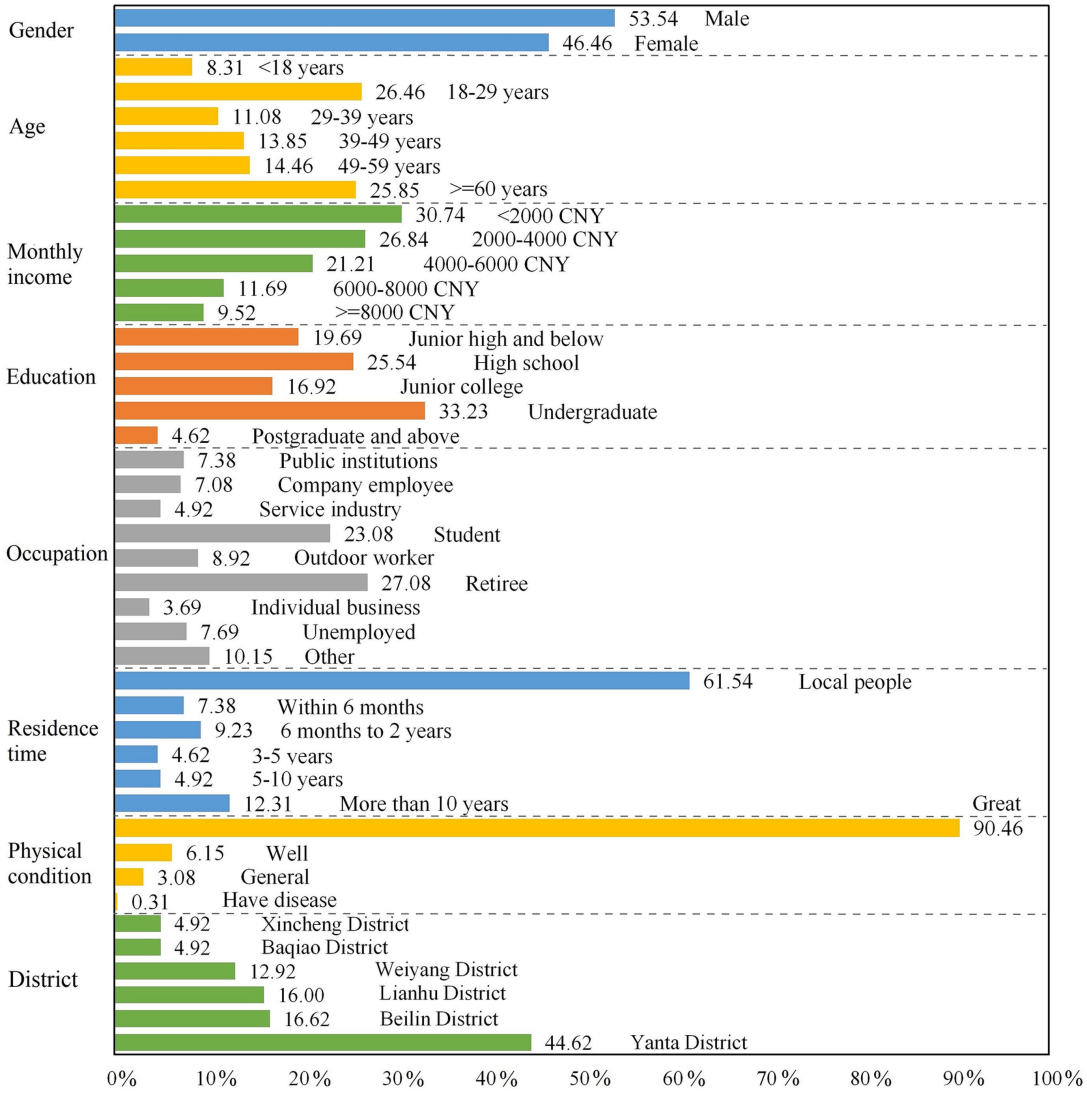
Basic information of the respondents.

Among the 325 valid questionnaires, there were 174 male and 151 female respondents, with a ratio of 1:0.87. In terms of age composition, young people aged 18–29 (26.46%) and older adult aged over 60 (25.85%) were, relatively, the largest groups. Corresponding to the age structure, retirees (27.08%) and students (23.08%) accounted for more than half of the occupations. The respondents with undergraduate education accounted for the largest proportion (33.23%), followed by high school (secondary specialized school or vocational–technical college) education (25.54%). More than 60% of the respondents were locals who have always lived in Xi’an, and more than half of them had a monthly income of less than 4,000 CNY. The respondents were generally in good physical condition, with 96.61% of the samples reported to be in great or in well condition. The basic attributes of the respondents are shown in Figure 2. Further, a stratification of the respondents’ basic information would be helpful to understand their sociodemographic characteristics. Both the respondents under 18 years old (male–female ratio = 2.00) and older adult over the age of 60 (male–female ratio = 1.80) were male-dominated, while, among young people aged 18–39 and middle-aged respondents aged 40–59, the proportion of female was slightly higher than that of male. Moreover, respondents’ health condition deteriorated gradually during the aging process. Among them, 96.30, 94.26, 92.39 and 80.95% of respondents under the age of 18, 18–39, 40–59 and over the age of 60 were in “great” physical condition, respectively. Judging from the gender structure, the proportion of male in the samples with “great” (53.06%) or “well” (65.00%) health conditions was higher than that of female, and the samples of “have disease” were all concentrated in female. From the perspective of age structure, the groups with “great” or “well” health conditions were concentrated in young people aged 18–39 (39.12%) and older adult over 60 years old (50.00%), respectively.

**Figure 3:**
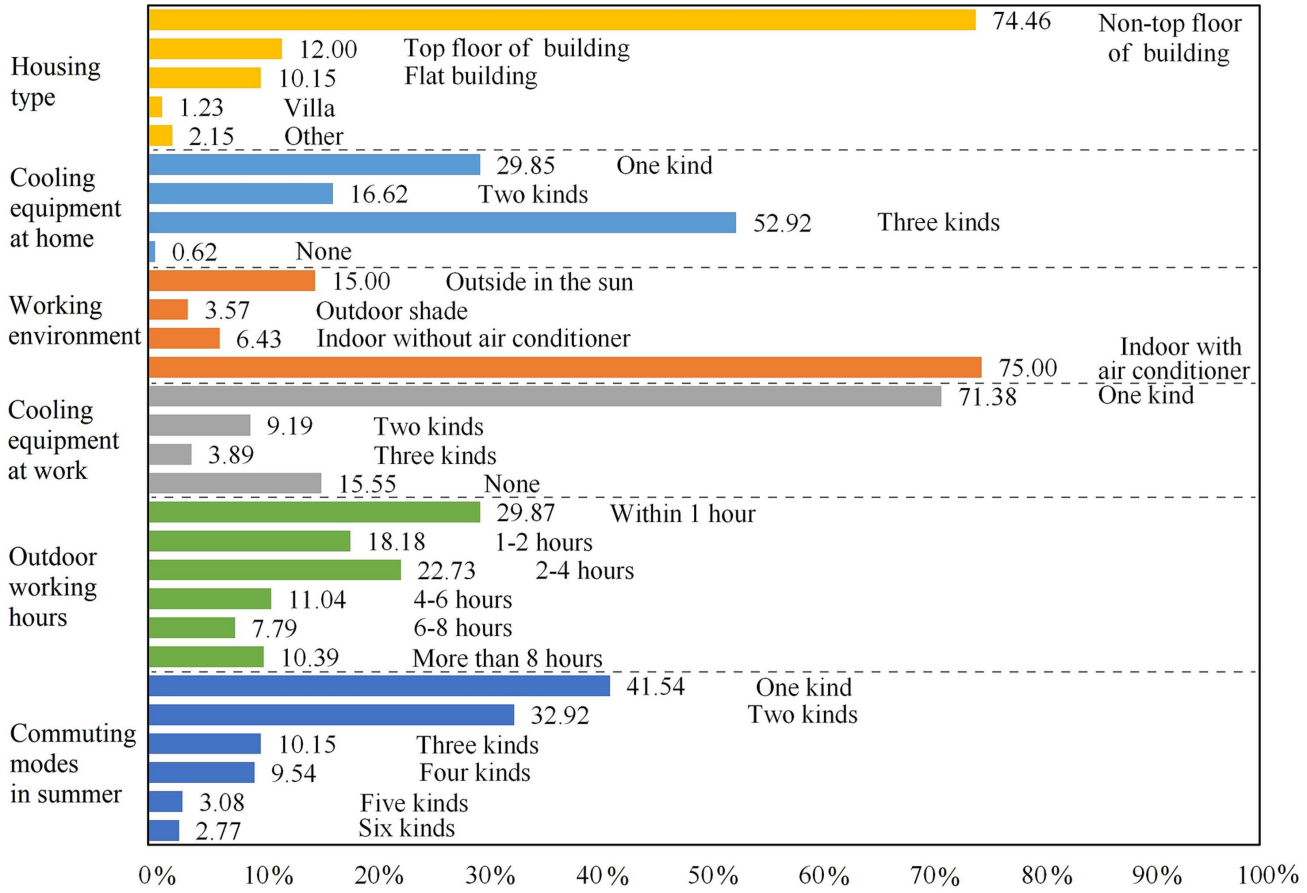
Living and working conditions of the respondents.

Differences in the urban residents’ working and living conditions, as well as in the cooling measures during summer and commuting modes were found to have a direct impact on respondents’ perceptions of hot days. The abovementioned characteristics of the respondents are summarized in Figure 3. In terms of living environment, the respondents mostly lived in a non-top floor of apartment buildings (74.46%), and the cooling equipment at home were mostly air conditioners, refrigerators, and fans. According to the survey, 52.92% of the respondents had all three kinds of cooling equipment, which were qualified to cope with the hot days; however, 0.62% of the respondents did not have any cooling equipment. The residents’ working environment could reflect their exposure characteristics in high temperature weathers. More than 70% of respondents worked in air-conditioned rooms and had at least one of the cooling equipment mentioned prior. Further, 48.05% of the respondents worked outdoors for less than 2 h per day in a relatively comfortable working environment. However, there were still 10.39% of them that were exposed to the outdoors for more than 8 h per day. In addition, residents generally used a combination of multiple modes of transportation to travel in summer. Respondents who only traveled by foot or by bicycle (i.e., electric bicycle), shaded public transports, and private cars accounted for 21.85, 12.92, and 6.77% of the total sample, respectively. In addition, the residents who traveled without shelter were mainly the older adult and retirees.

### 3.2. Difference analysis and influencing factors of the health risk perceptions of hot days

#### 3.2.1. Difference analysis of the health risk perceptions of hot days

**Figure 4:**
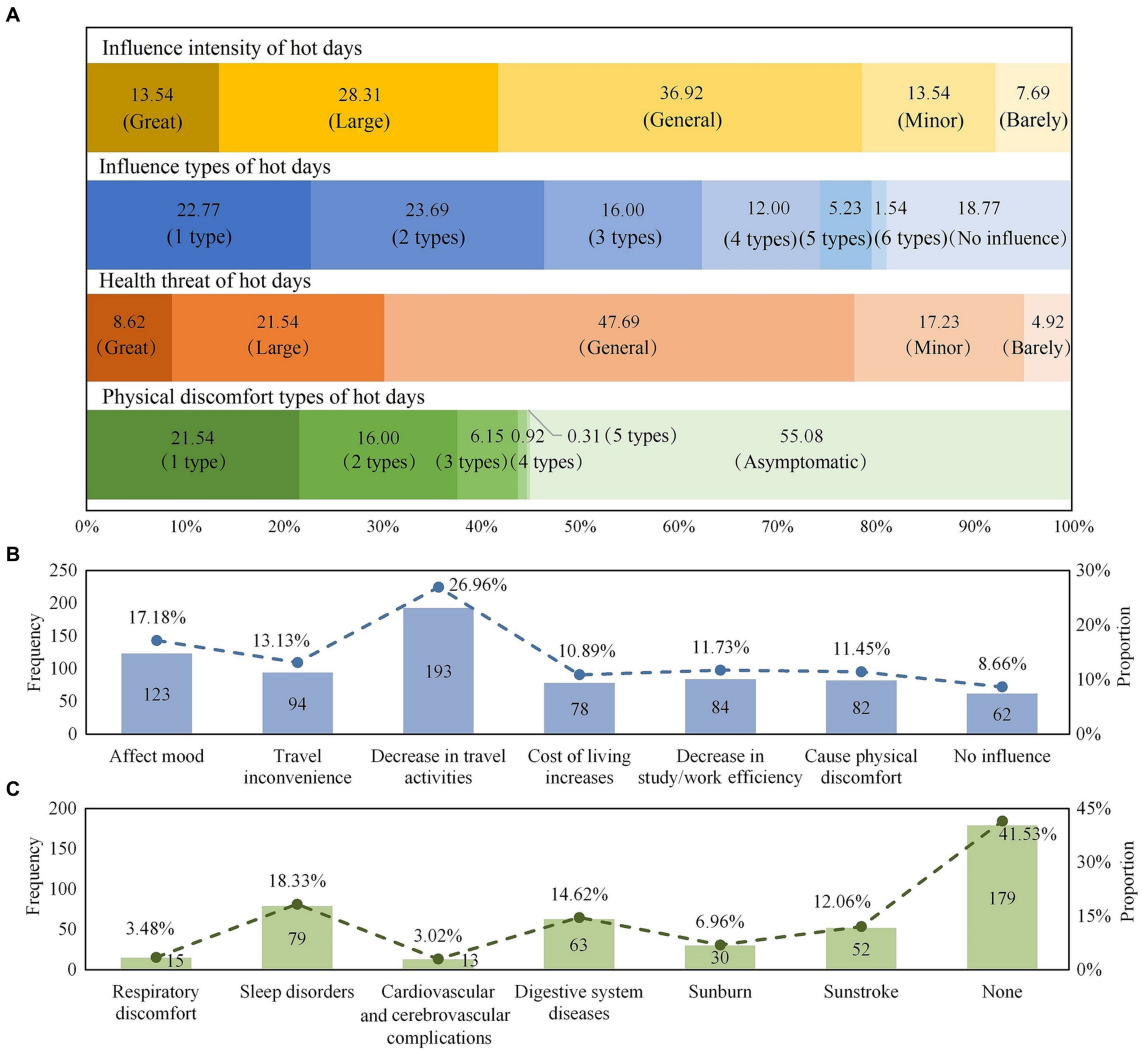
**(A)** Health risk perceptions of hot days, **(B)** the frequency of influence types of hot days, and **(C)** the frequency of physical discomfort types due to hot days.

Clarifying the urban residents’ health risk perceptions of hot days and the differences among the different people groups would help to take appropriate assistance measures for specific groups, thereby reducing the health risks of heat exposure. According to the survey, the respondents thought that the high temperature weather was mainly within the range of 24–39°C; having said that, 66.15% of them thought that the hot days were above 35°C. At the same time, 45.85% of the respondents believed that the hot days were concentrated in July and August, and there were also certain residents who said that there was a tendency for them arrive in advance to June and were then postponed in September. The residents’ health risk perception of hot days could be detected from four dimensions (Figure 4A), 92.31% of the residents believed that the high temperature weathers in Xi’an had an impact on themselves (including 13.54% for “great influence,” 28.31% for “large influence,” 36.92% for “general influence,” and 13.54% for “minor influence”). Further, 81.23% of the respondents were disturbed to varying degrees in their daily work and life, and 58.46% of them were affected by at least two kinds of disturbance. Specifically, the influence types of hot days were mainly in the form of reduced travel frequency (26.96%), mood swings (17.18%), travel inconvenience (13.13%), decreased study or work efficiency (11.73%), physical discomfort (11.45%), and increased living costs (10.89%) (Figure 4B). Nearly half of the respondents had a moderate perception of the health threat posed by hot days (47.69%), but still over 30% of the respondents declared “great health threat” (8.62%) and “large health threat” (21.54%). Moreover, 44.92% of the respondents had medical treatments due to hot days, among which the frequency of sleep disorders (18.33%), digestive system discomfort (14.62%), or sunstroke (12.06%) was relatively high, some of the respondents showed the symptoms of sunburn (6.96%), respiratory system discomfort (3.48%), and cardiovascular and cerebrovascular complications (3.02%) (Figure 4C).

In this study, the ANOVA in SPSS 25.0 was used to explore the different characteristics among the multiple people groups who are affected by hot days. The basic information and the living or working conditions of the respondents were taken as the independent variables, and their health risk perceptions of hot days were taken as the dependent variables, respectively. With respect to the aforementioned, the test standard was *p* < 0.05. In terms of the group differences in Table 2, urban residents with different working environments and cooling equipment at work showed significant differences in regard to the influence intensity of hot days on their daily lives. Meanwhile, there was no statistical difference found among the other factors. According to the mean value of the dependent variable, it could be seen that people who work outdoors could feel the influence of hot days more strongly than those who worked indoors. Among them, the people who worked in the outdoor shade had the highest perception degree (Mean = 3.73, S.D. = 1.01), and those who worked indoor with air conditioners were the least affected by hot days (Mean = 3.17, S.D. = 1.07). In addition, the respondents’ perceptions of the influence intensity of hot days decreased with the increase in the number of cooling equipment in the workplace. Those with three kinds of cooling equipment (Mean = 2.46, S.D. = 1.29) were significantly less affected by hot days than those with no cooling equipment (Mean = 3.61, S.D. = 1.33), or those who were with only one (Mean = 3.50, S.D. = 1.03) or two kinds (Mean = 3.21, S.D. = 0.99).

**Table 2:** Differences in the health risk perceptions of hot days for different people groups.

Perception	Category	Type	Mean	S.D.	*F*	*p*
Influence intensity of hot days	Working environment	Outside in the sun	3.59	1.22	2.65	0.05
		Outdoor shade	3.73	1.01		
		Indoor without air conditioner	3.35	1.05		
		Indoor with air conditioner	3.17	1.07		
	Cooling equipment at work	None	3.61	1.33	4.19	0.01
		One	3.50	1.03		
		Two	3.21	0.99		
		Three	2.46	1.29		
Influence types of hot days	District	Yanta District	1.43	0.77	2.65	0.02
		Beilin District	1.46	0.72		
		Lianhu District	1.23	0.81		
		Weiyang District	1.50	0.80		
		Baqiao District	0.88	0.96		
		Xincheng District	1.69	0.60		
	Cooling equipment at work	None	1.69	0.83	2.84	0.04
		One	1.40	0.78		
		Two	1.32	0.55		
		Three	0.91	0.94		
Health threat of hot days	Gender	Male	2.86	0.98	28.76	0.001
		Female	3.41	0.84		
	Working environment	Outside in the sun	3.50	1.03	3.16	0.03
		Outdoor shade	3.27	1.19		
		Indoor without air conditioner	3.11	0.98		
		Indoor with air conditioner	3.04	0.92		
	Outdoor working hours	Within 1 h	2.67	1.03	17.84	0.001
		1–2 h	3.08	0.70		
		2–4 h	3.05	0.66		
		4–6 h	3.17	0.86		
		6–8 h	3.72	1.00		
		> 8 h	4.19	0.82		
Physical discomfort types due to hot days	Gender	Male	0.55	0.78	10.56	0.001
		Female	0.84	0.86		

It could be found from Table 2 that residents in the different districts showed significant differences in regard to the number of hot days’ impact on their daily lives. Since the eastern part of the Baqiao District is mountainous, the urban heat island and threat of hot days are relatively limited. As such, the residents in this district were affected by the least amount of influence types of hot days (Mean = 0.88, S.D. = 0.96). Meanwhile, the more cooling equipment the respondents had at their workplace, then the less they were affected by the specific influence types of hot days. Hot days has widely known as an important risk for death and disease related factors, in this study, the residents from different gender, working environment and duration of outdoor work showed significant differences in their perceptions of the health threat caused by hot days. Women (Mean = 3.41, S.D. = 0.84) were more likely to feel the health threat from hot days than men (Mean = 2.86, S.D. = 0.98); respondents who worked indoors with air conditioner (Mean = 3.04, S.D. = 0.92) were less exposed to health threat from hot days than those who worked outdoors with (Mean = 3.27, S.D. = 1.19) or without shade (Mean = 3.50, S.D. = 1.03), and as the increasing of outdoor working hours, residents’ perception of health threat from hot days enhanced significantly. Most of the respondents who worked outdoors for more than 8 h felt that they were exposed to “large” or “great” health threats, while those who were exposed to outdoors for less than 1 h generally felt a “general” or “minor” health threat. Moreover, gender also showed a significant difference in regard to the specific medical treatment due to the impact of hot days for different people groups. Among the various types of physical discomfort, women (Mean = 0.84, S.D. = 0.86) had more symptoms and showed a higher vulnerability than men (Mean = 0.55, S.D. = 0.78).

#### 3.2.2. Influencing factors of the health risk perceptions of hot days

**Figure 5:**
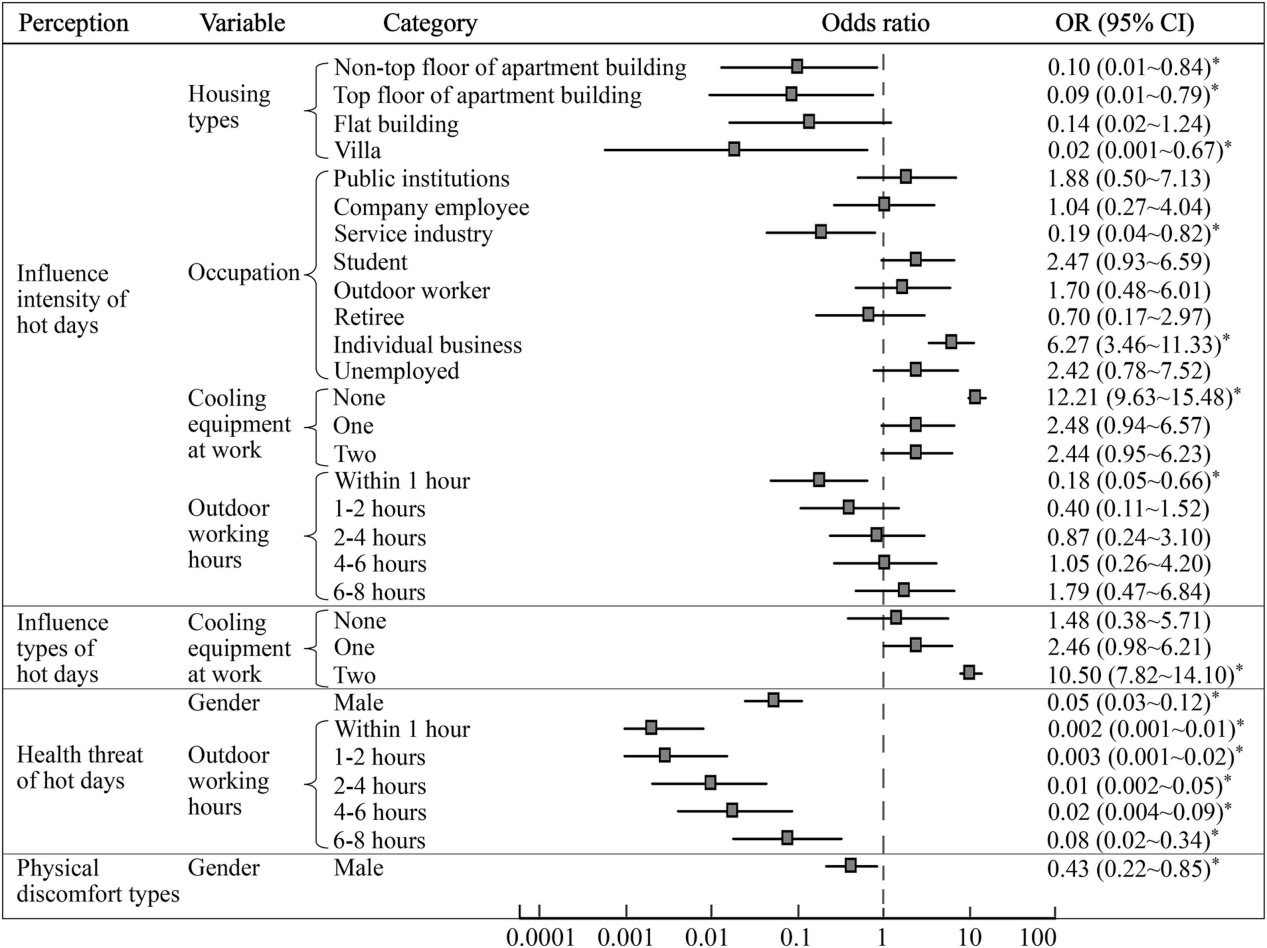
Influencing factors of health risk perceptions of hot days (* means *p* < 0.05).

With respondents’ basic information and living or working conditions as the independent variables, and their health risk perceptions of hot days as the dependent variables, the logistic regression model regarding the influencing factors of residents’ perceptions of hot days was built (Figure 5). The results showed that housing types, occupation, cooling equipment at work, and outdoor working hours significantly affected residents’ perceived intensity of the influence of hot days. Specifically, people living on a non-top floor (*OR* = 0.10, 95% *CI*: 0.01 ~ 0.84, *p* = 0.03) or top floor of apartment building (*OR* = 0.09, 95% *CI*: 0.01 ~ 0.79, *p* = 0.03), and villas (*OR* = 0.02, 95% *CI*: 0.001 ~ 0.67, *p* = 0.03) were less likely to have stronger perceptions of the threat of hot days than those living in other environments (such as prefabricated houses). In addition, the likelihood of residents in the service industry and individual businesses received a strong influence intensity of hot days was found to be 0.19 times and 6.27 times higher in comparison to other occupations (such as freelancers, farmers, etc.), respectively. This fact was deemed to be related to the outdoor exposure time in the different occupations. Respondents without any cooling equipment in the workplace perceived a much higher influence intensity of hot days than those with three kinds (*OR* = 12.21, 95% *CI*: 9.63 ~ 15.48, *p* = 0.01). Residents who worked outdoors for less than 1 h in summer were less likely to perceive the threat of hot days than those who worked outside for more than 8 h (*OR* = 0.18, 95% *CI*: 0.05 ~ 0.66, *p* = 0.01). In terms of the specific impact of hot days, residents with two kinds of cooling equipment at work were more affected than those with three kinds (*OR* = 10.50, 95% *CI*: 7.82 ~ 14.10, *p* = 0.04), which indicating that the combined use of multiple cooling equipment could significantly alleviate the thermal threats on the residents’ daily lives.

Figure 5 shows that gender was a significant influence factor both on the health threat and the discomfort symptoms caused by hot days. Men declared they experienced less health threat than women (*OR* = 0.05, 95% *CI*: 0.03 ~ 0.12, *p* = 0.001), in other words, women were 18.52 times more likely than men to suffer health threats from hot days. Meanwhile, men were less likely to seek medical treatment and had fewer symptoms (*OR* = 0.43, 95% *CI*: 0.22 ~ 0.85, *p* = 0.02), which is consistent with the findings in Table 2. Long-term exposure to hot and humid environments could make the ambient temperature exceed the acceptable limit of human, which could directly cause heat-related diseases (i.e., heat cramps, heat exhaustion and heat stroke) (35). This study found that the outdoor working hours significantly affected the perception of heat threat (*p* < 0.05), the longer the respondents worked outdoors during the daytime in summer, the higher their perceived health risks from heat exposure.

### 3.3. Difference analysis and influencing factors of the perceptions of cooling effect

#### 3.3.1. Difference analysis of the perceptions of urban ecological landscape’s cooling effect

**Figure 6:**
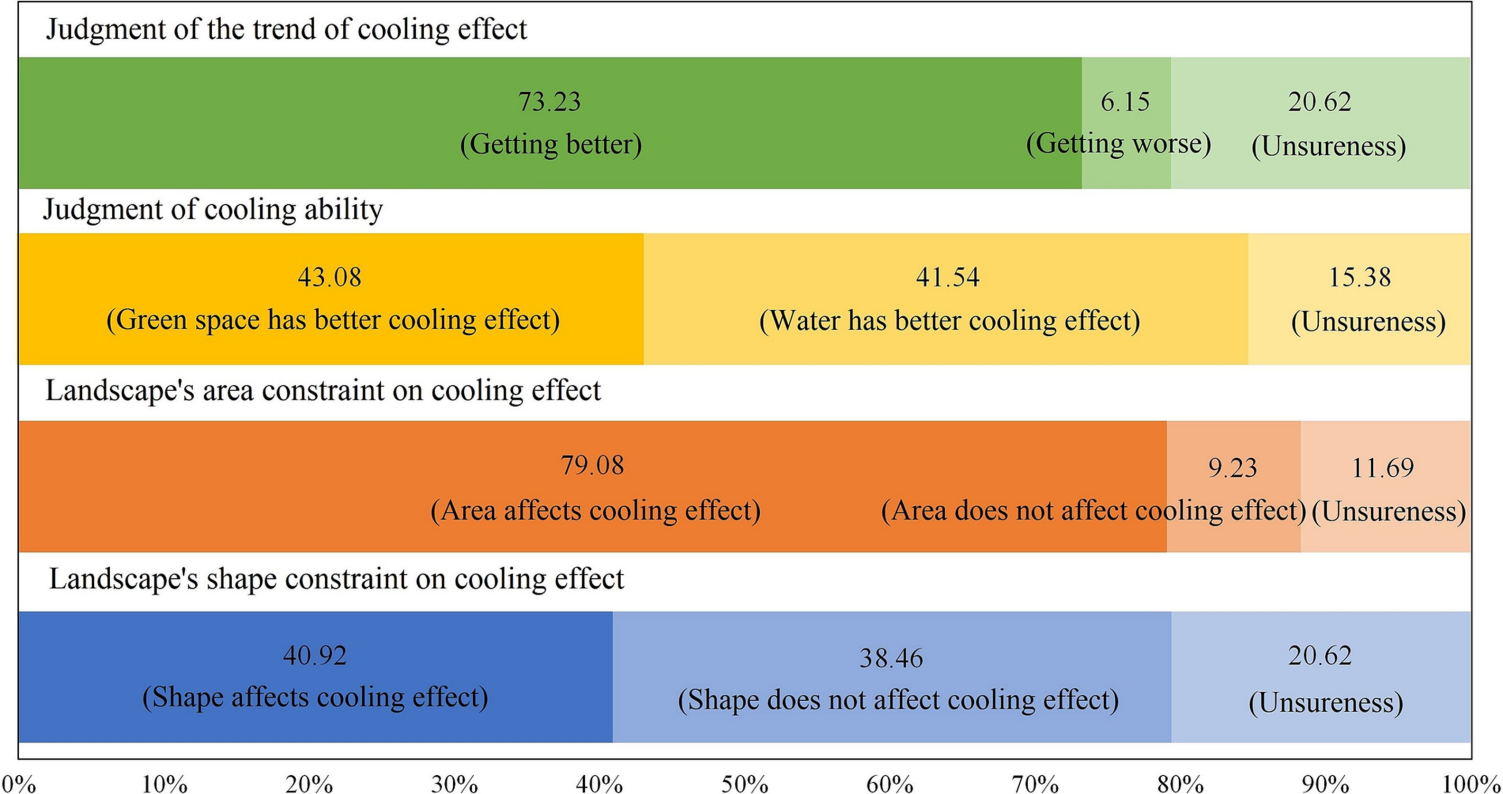
Perceptions of the cooling effect of urban green spaces and water landscapes.

In this study, the difference in the perception of the landscape’s cooling effect was analyzed from the judgment of the trend and ability of the cooling effect, as well as with respect to the impact of the landscape pattern on the cooling effect. The basic information, the living or working conditions of the respondents and their perceptions of the cooling effect were taken as the independent variables and dependent variables for the ANOVA, respectively. The survey showed that most of the respondents were optimistic about the trend of the cooling effect of urban green spaces and water landscapes in Xi’an in recent years (73.23%). However, 20.62% of the residents could not make a clear judgment, and the remaining believed that the cooling effect has diminished (Figure 6). However, there was no statistical difference in the judgment of cooling trend for different people groups through the ANOVA. In terms of judging the difference in the cooling ability between urban green spaces and water landscapes, as can be seen from Figure 6, the proportion of respondents who believed that the cooling effect of urban green spaces is better (43.08%) was only slightly higher than that of who thought water has a stronger cooling ability (41.54%). Table 3 shows that people of different ages had significant differences in judging the cooling effect of urban green spaces and water landscapes. Combining the mean value and the assignment rule of the dependent variable (variable assignment: 1 = green space has better cooling effect, 2 = unsureness, and 3 = water has better cooling effect), it could be seen that people under 30 years old were more inclined to believe that water bodies have a stronger cooling ability (18–29 years old: Mean = 2.43, S.D. = 0.68; <18 years old: Mean = 2.56, S.D. = 0.58). This is related to the fact that the young group always has a wider access to more relevant knowledge and more relevant education, such that their judgment on the ability of the landscape’s cooling affect is more accurate.

**Table 3:** Differences in the perceptions of the cooling effect for different people groups.

Perception	Category	Type	Mean	S.D.	*F*	*p*
Judgment of cooling ability	Age	<18 years old	2.56	0.58	3.14	0.01
		18–29 years old	2.43	0.68		
		30–39 years old	2.06	0.67		
		40–49 years old	2.18	0.68		
		50–59 years old	2.15	0.66		
		≥60 years old	2.19	0.78		
Landscape’s area affects cooling effect	Education	Junior high school and below	1.52	0.82	3.88	0.01
		High school	1.53	0.74		
		Junior college	1.33	0.61		
		Undergraduate	1.25	0.62		
		Postgraduate and above	1.17	0.44		
	Cooling equipment at work	None	1.52	0.76	2.79	0.04
		One	1.29	0.62		
		Two	1.12	0.33		
		Three	1.18	0.60		
Landscape’s shape affects cooling effect	Age	<18 years old	2.00	0.88	2.83	0.02
		18–29 years old	1.69	0.82		
		30–39 years old	2.00	0.91		
		40–49 years old	2.06	0.91		
		50–59 years old	2.17	0.89		
		≥60 years old	2.17	0.91		

The respondents had different opinions on whether landscape patterns affected the cooling effect. Residents had a higher acceptance (79.08%) for the restrictive effect of the area of urban green spaces and water landscapes regarding its cooling effect, while only 40.92% of residents believed that the shape of the landscape also had an impact, and another 38.46% held the opposite attitude (Figure 6). In addition, the respondents were invited to rate, on a scale of 0 to 5, the cooling ability of ecological landscapes among different shapes (the higher score means a better cooling ability). The scoring order was in terms of irregular shape (3.76), roundness (3.37), rectangular (3.27), and foursquare (3.08), which is consistent with the results of existing studies, whereby it was found that the more complex the shape of green patches inside a park was then the wider the influence of the cooling effect (36).

The difference analysis in Table 3 shows that residents with different education levels and cooling equipment at work had significant differences in their perceptions of whether the landscape’s area would impact its cooling effect. Among them, highly educated people showed a higher degree of recognition of the restrictive effect of landscape area on the cooling effect. At the same time, residents with more types of cooling equipment in the workplace were more inclined to accept that changes in the area of urban parks would affect their cooling effect. It can be seen from Table 3 that residents from different ages had significantly different perceptions of whether the landscape’s shape would impact its cooling effect. In addition, their perceptions showed a trend of increasing first and then weakening with older age. Among them, the group who mostly recognized that the shape of the landscape would affect its cooling effect was concentrated in the 18–29 years old group (Mean = 1.69, S.D. = 0.82). Meanwhile, the middle-aged (30–39 years old: Mean = 2.00, S.D. = 0.91; 40–49 years old: Mean = 2.06, S.D. = 0.91) and older adult (50–59 years old: Mean = 2.17, S.D. = 0.89; ≥60 years old: Mean = 2.17, S.D. = 0.91) were not sensitive enough to the influence of this topic.

#### 3.3.2. Influencing factors of the perceptions of urban ecological landscape’s cooling effect

**Figure 7:**
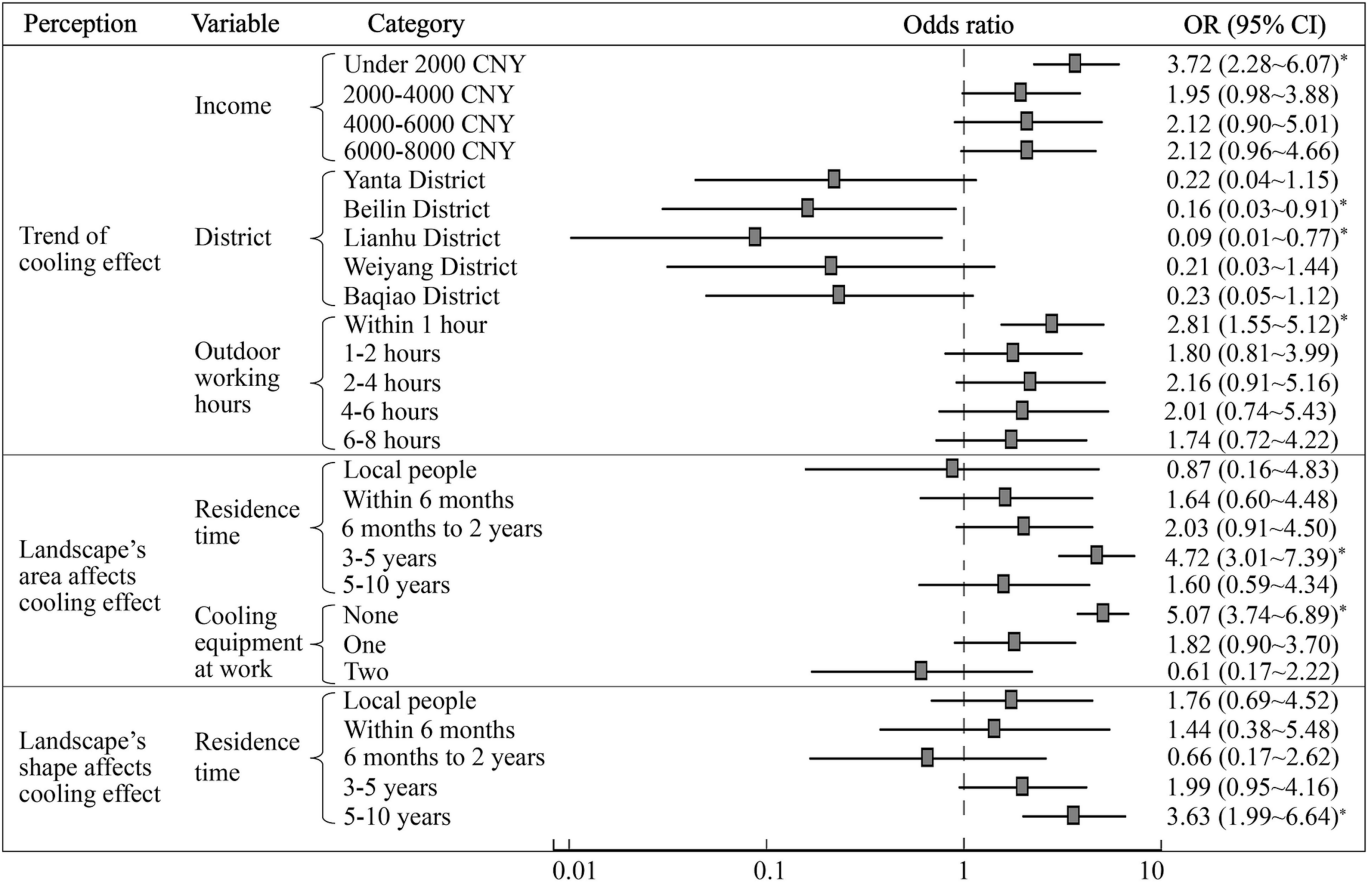
Influencing factors of perceptions of the urban ecological landscape’s cooling effect (* means p < 0.05).

Figure 7 shows the ordinal logistic regression model regarding the influencing factors of residents’ perceptions of urban ecological landscape’s cooling effect. It should be noted that respondents’ judgment of cooling ability was not taken considered because it is not a continuous variable. It can be seen that residents’ monthly income, district, and outdoor working hours had a significant impact on the judgment of the urban parks’ cooling trend in Xi’an. Specifically, respondents with a monthly income of less than 2000 CNY were more likely to believe that the cooling effect of parks was getting worse than those with a monthly income of more than 8,000 CNY (*OR* = 3.72, 95% *CI*: 2.28 ~ 6.07, *p* = 0.02). In addition, who worked outdoors for more than 8 h per day in summer were more likely to believe that the cooling ability of urban parks was enhancing than those who were exposed outdoors for less than 1 h (*OR* = 2.81, 95% *CI*: 1.55 ~ 5.12, *p* = 0.04). This is because for workers who were always exposed to the outdoors in summer, green parks were important shelters for them when facing the threat of hot days (37), so they are more sensitive to the strengthening of the construction of urban green infrastructure. From a spatial perspective, residents in the Xincheng District showed the most pessimistic attitude towards the cooling trend of urban parks. Respondents in the Beilin District (*OR* = 0.16, 95% *CI*: 0.03 ~ 0.91, *p* = 0.04) and Lianhu District (*OR* = 0.09, 95% *CI*: 0.01 ~ 0.77, *p* = 0.03) were less likely than those in the Xincheng District to think that the cooling effect was getting worse.

The residence time in Xi’an and the number of cooling equipment at work had significant constraints on the perception of whether the landscape’s area affected the cooling effect. Among them, residents who have lived in Xi’an for more than 10 years were more inclined to give a positive answer to this question than those who had only settled down with the last 3–5 years (*OR* = 4.72, 95% *CI*: 3.01 ~ 7.39, *p* = 0.04). In addition, respondents without any cooling equipment at work were 5.07 times more likely to disagree that landscape’s area had effect on their cooling effect than those who had three cooling devices (95% *CI*: 3.74 ~ 6.89, p = 0.04). It can be seen from Figure 7 that residents who have lived in Xi’an for 5–10 years were less likely to agree with the constraint of the landscape’s shape on the cooling effect than those who have lived in Xi’an for more than 10 years. In addition, the probability that a respondent thought that the shape “has no impact” was 3.63 times than that of the latter (95% *CI*: 1.99 ~ 6.64, *p* = 0.03). In conclusion, residents with a longer period of residency had a more accurate understanding of the cooling ability of urban green spaces and water landscapes under the influence of landscape patterns.

### 3.4. Impact of health risk perception on residents’ needs of urban ecological construction

**Figure 8:**
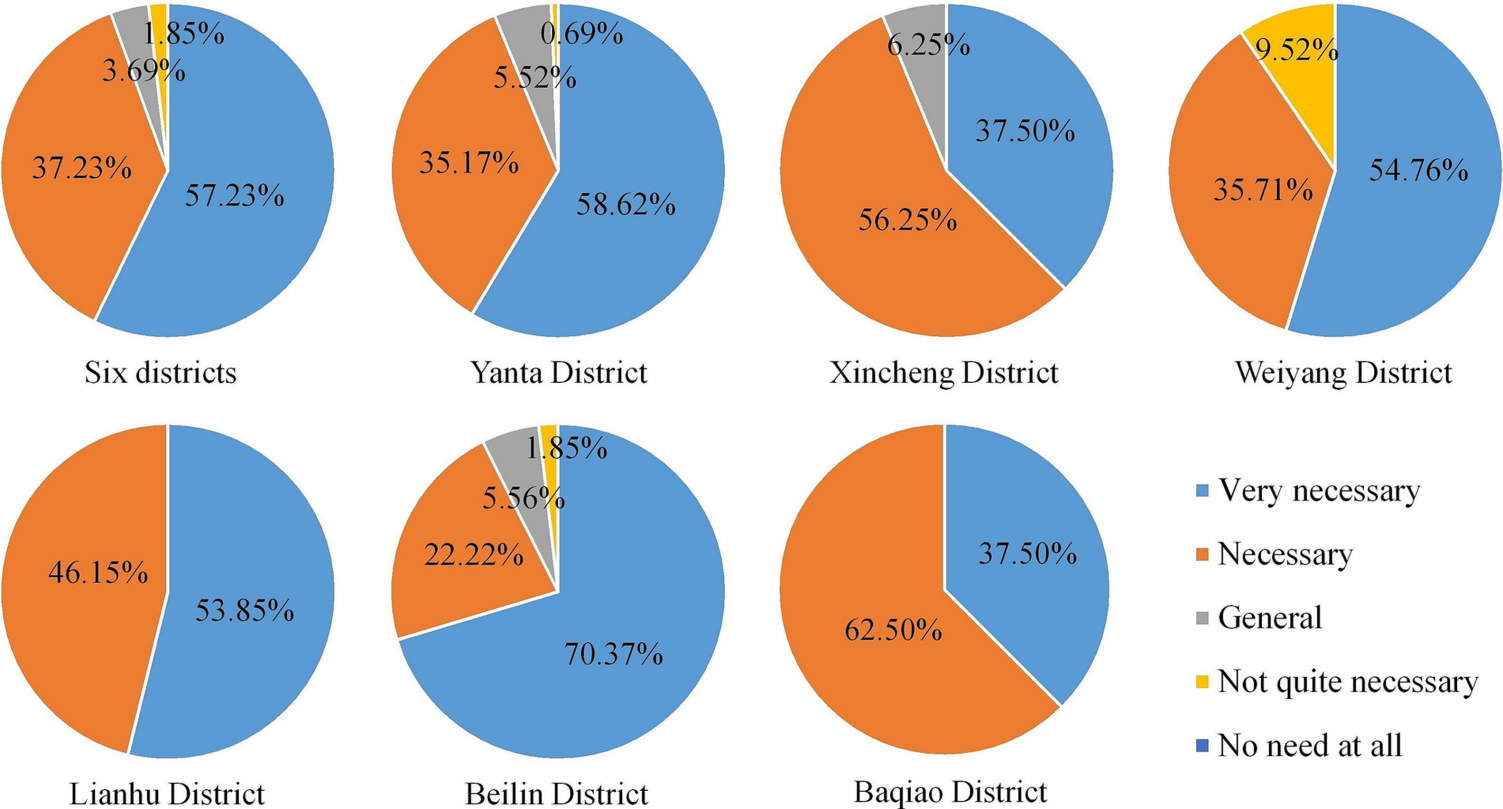
Necessity of urban green spaces and water landscapes construction.

Identifying the needs and suggestions of the respondents on urban green infrastructure could improve urban residents’ ability to prevent heat risks. In the face of hot weather, residents hoped that the government would take countermeasures, mainly including those related to guaranteeing water and power supply (18.36%), supplementing street trees (18.20%), increasing green infrastructure (16.44%), obtaining the high temperature warnings timely (11.44%), and replenishing air-conditioned public cooling spaces (10.75%). A relatively small number of respondents wanted to adjust working hours in summer, provide heat subsidies to needy families, and provide heatstroke prevention medicines. The results showed that more than 90% of the respondents could recognize the importance of the ecological landscape in mitigating urban thermal risks, and 57.23% of them believed that the construction of more urban green spaces is particularly necessary. More than 50% of the respondents in the Yanta, Weiyang, Lianhu, and Beilin districts were in urgent need for urban ecological landscape construction (Figure 8), among which the residents in the Beilin District thought were “very necessary” accounted for the highest proportion (70.37%).

**Figure 9:**
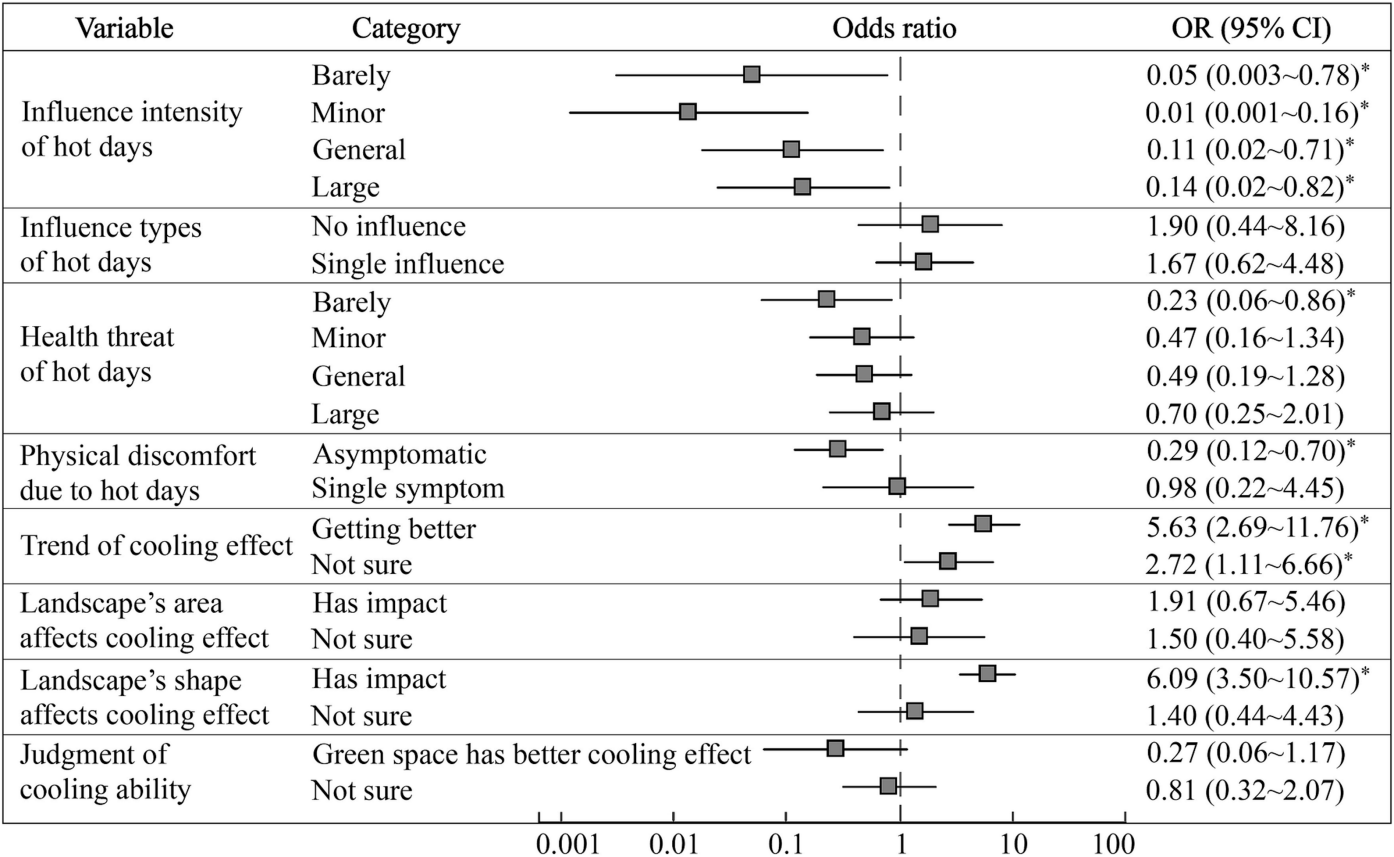
Influencing factors of residents’ needs of the urban green spaces and water construction (* means *p* < 0.05).

Residents’ needs of urban green spaces and water construction is closely related to their cognition and adaptation to the thermal environment. By taking their ratings on the necessity of green cities as the dependent variable, and their health risks perception from hot days and their understanding of the cooling effect of existing ecological landscapes as the independent variables, an ordinal logistics model between these indicators was built. It could be found that the stronger the perception of influence intensity of hot days, the greater the respondents’ needs of the construction of urban green spaces (Figure 9), which was statistically significant (*p* < 0.05). However, the specific impact they received did not significantly related to their needs. In addition, this study found that compared with the respondents who experienced great health threats or who with various physical symptoms due to hot days, residents who thought they were barely threatened (*OR* = 0.23, 95% *CI*: 0.06 ~ 0.86, *p* = 0.03) or without any discomfort (*OR* = 0.29, 95% *CI*: 0.12 ~ 0.70, *p* = 0.05) showed significantly lower needs of urban green spaces construction. Figure 9 shows that residents who were optimistic or uncertain about the urban green spaces’ cooling trend in Xi’an, their needs of the construction of green infrastructure was 5.63 times and 2.72 times stronger than those who believed that the cooling effect had a worsening trend. Respondents with higher cognition on the constraining effect of landscape pattern on its cooling effect were also more inclined to strongly support urban ecological construction. However, the judgment of the cooling ability between green spaces and water did not affect whether they supported urban ecological construction.

Among the specific suggestions for the urban ecological construction, the respondents generally hoped to increase the number and range of green infrastructure, as well as desired to improve the practicality and ornamental value of green belts. Thus, while providing shade for residents’ outdoor activities, these measures could also establish a green image for the city. In terms of urban water construction, the respondents suggested strengthening the layout optimization and water quality of rivers and lakes in Xi’an, especially proposed to strengthen the manual management of urban green spaces around water bodies, the existing research has provided that the cooling effect of water was affected by the proportion of surrounding green spaces, which could produce synergistic cooling effect (38). By doing this, the aesthetics and practicality of urban water bodies in terms of truly developing a cooling effect via the water landscapes could be combined.

## 4. Discussion

### 4.1. Comparison with existing studies

In terms of residents’ health risk perceptions of hot days, this study found that residents with a relatively poor living environment were likely to have stronger perceptions of the threat of hot days, in other words, they could be more vulnerable to hot days, which was consistent with the existing research (39). The living environment is an intuitive reflection of a residents’ income, and is closely related to the cooling measures taken in the face of heat waves, as well as in their ability to purchase cooling equipment at home, which, in turn, has a significant constraint on their high temperature perception. In addition, this study found that residents’ educational background also significantly constrained their cognition, which has also been confirmed in existing studies. Toan et al. noted that for people living in slums and non-slum areas, there was a positive correlation between the education of respondents and their perceived influence level of hot days (40). Furthermore, significant gender differences in residents’ exposure to high-temperature health risks was found in this study, this phenomenon was also been proved. Yu et al. found that the effects of high temperature on mortality were modified by gender, of which the harmful effect among women was more than 20 times that among men (41). Therefore, special attention should be paid to the health of women, especially pregnant women and young children under the threat of high temperature risks.

Exploring residents’ perceptions, needs and suggestions on the cooling effect of urban green spaces and water bodies is one of the important solutions for urban thermal risk prevention. This study found that, compared with water bodies, the proportion of residents who believed that green space had a better cooling ability was relatively higher. While, existing studies have shown that the cooling amplitude and cooling intensity of water was more advantageous than that of urban green spaces within the same area (42). The major reasons for this perception bias are the relative lack of urban natural water and the small area of water bodies in urban parks in Xi’an City, as well as the fact that the respondents answered without a clear cognition of this question. Existing studies have proved that there was a nonlinear correlation between landscape patterns and urban heat islands (43), and the mechanism of landscape’s shape on its cooling effect was more complex than that of area (44). However, the respondents in this study still showed a lack of awareness regarding this phenomenon, which reflected the necessity of strengthening the popularization of relevant knowledge and to the urgency of further deepening the research on the correlation mechanism between landscape patterns and urban thermal environments.

Duan et al. declared that the users of urban green infrastructure generally believed that the high temperature and health risk could be mitigated by urban green spaces (45). At the same time, urban residents prefer multi-functional urban green spaces (46), and the lack of green landscapes in the living environment will increase their willingness to relocate (47). At present, residents’ thermal risk perception has attracted much attention, but one of the goals of which is to guide urban ecological construction and create a better settlement environment. Meanwhile, urban planning in rapidly urbanizing areas is still mainly guided by the needs of socioeconomic development, and the limited ecological land puts forward higher requirements for reasonable planning oriented to residents’ perceptions and suggestions. Based on the research on the thermal risk perception of urban residents, this study expanded the analysis of their perceptions of urban ecological construction, which promoted the comfort of urban residents to get more attention, and also provided a reference for the development of related research.

### 4.2. Interaction between air pollution and hot days

With the continuous acceleration of urbanization, air pollution has become another important factor threatening residents’ health and urban environment besides hot days. Typical urban air pollutants include PM2.5, SO_2_, NO_2_, and O_3_, for every 10 μg/m^3^ growth of which, the mortality rate of people from non-accidental and cardiovascular diseases would increase significantly (48). Xi’an City has a subhumid continental monsoon climate and is located in the plain area, where air pollutants are not easily dispersed in winter and spring. Moreover, with the coal as an important local energy consumption, frequent air pollution incidents have occurred in Xi’an in recent years. A study in Xi’an found that for every 10 μg/m^3^ increase in short-term atmospheric PM10, SO_2_, and NO_2_ concentrations, the number of outpatient visits for schizophrenia increased by 0.29, 1.37, and 1.88%, respectively (49). In terms of residents’ perceptions, Orru et al. built a model that describes interrelations between air pollution, health risk perception, health symptoms and diseases (50), and Noël et al. carried out a review analysis of the qualitative research about public health risk perceptions on air pollution (51).

Previous studies have shown that there is a close interaction between urban thermal environment and urban air pollution. Among them, the temperature difference between the central urban area and the suburbs affects the atmospheric circulation, transport and deposition of pollutants, resulting in the reduction of air pressure and the backflow and accumulation of pollutants (52). At the same time, regional differences in pollutant concentrations would also affect the internal radiation of cities, thereby restricting the intensity and pattern of the thermal environment (53). The interaction between them is also an important factor leading to the increase of diseases and mortality of urban residents. A study on 9 European cities pointed out that high temperature caused more deaths when PM10 concentration increased (54), and Schaefer et al. (55) combined the investigation of pedestrian perception on local heat stress and air quality as well as the remote sensing data, and analyzed the regulation path of local microclimate (55). Air pollution was not considered in the questionnaire, which is the limitation of this study. Considering the typicality of urban thermal environment and air pollution in Xi’an, combining the interaction of the two on residents’ health perception and their adaptations would be the focus of future researches.

### 4.3. Impact of questionnaire design on research results

At present, questionnaire survey has become an important approach to carry out researches on health risk perception of urban residents. Questionnaire design is a comprehensive reflection of interviewers’ research objectives, and whether the structure and question setting are reasonable would also have a direct impact on the interpretation of the results. According to the difference in question setting, the questionnaire could be divided into three categories. Among them, the closed-ended questionnaire means that the respondents could only choose from the listed answers. It is conducive to the respondents’ effective and rapid response, reduces the time cost, and has obvious advantages in the statistical analysis and summary of the results (56). However, the closed-ended question also makes it difficult for respondents to exert their subjective initiative effectively, and would be easy for them to answer some complicated questions randomly. In contrast, open-ended questionnaires do not set standard answers, and respondents could answer according to their own understanding. It has the advantage of being flexible and diverse, which is conducive to obtaining the innovative thoughts of the respondents. However, open-ended questionnaires are difficult to analysis quantificationally and usually time-consuming, respondents tend to be reluctant to think deeply due to boredom, thereby reducing the validity of the questionnaires (57). Hybrid questionnaire makes up for the shortcomings of them, questions that could be answered clearly are usually set as closed-ended, while innovative questions (e.g., suggestions and opinions) are mainly open-ended.

The questionnaire used in this study is of a hybrid type. Among them, most of the objective questions (e.g., basic information, working and living conditions) were closed-ended. For the perceptual questions that required a description of the degree, the respondents were invited to choose in the form of a five-level Likert scale. At the same time, questions about the specific temperature and duration of hot days, the scores of cooling effect for urban green space in different shapes, and the needs and suggestions were open-ended. In addition, this study also set up some hybrid questions to help respondents to supplement the answers that were not considered during the questionnaire design. It should be pointed out that since the field survey in this study was carried out during the hot days in summer, the number of open-ended questions was controlled considering that the respondents tended to refuse to answer due to the high temperature. However, understanding the diverse thoughts of urban residents is pivotal to improve the depth of research.

Therefore, in the further studies, the investigators would adjust the questionnaire design and increase the number of open-ended questions based on this survey. In order to avoid the boredom of the respondents, a combination of recording and keyword response could be used. Specifically, for respondents who are unwilling to directly fill in the open-ended questions, they can be invited to give an oral account of their opinions, and the investigators would make a recording them for subsequent analysis after obtaining their consent. The keyword response is to invite the respondents to fill in several keywords generated by their first reaction, so as to depict a visual map of the respondents’ thoughts based on the big data technology. At the same time, the residents’ health risk perception due to hot days in Xi’an is a topic worthy of long-term tracking. The regular investigations and the constantly optimization of the questionnaire design would be conducted in further studies, so as to establish a continuous and dynamic investigation database.

### 4.4. Limitations

Based on the perceptions of residents against the background of urban thermal environments, this study highlighted the direct needs of urban residents, which could effectively support urban planning for the improvement of the quality of living environments. However, there is still room for further improvements in this study. In terms of data and sample selection, due to manpower constraints in the survey, the samples only covered 13 typical urban parks in Xi’an City. In a further study, more comprehensive results could be obtained by expanding the survey scale. At the same time, the construction of ecological landscapes is an effective and necessary approach by which to alleviate hot days. The cooling effect of urban parks is closely related to climate change, urban green infrastructure planning, and relevant government policies (58). Residents’ perceptions of hot days and the cooling effect of landscapes will also change dynamically. Therefore, the long-term monitoring should be the future direction of this study, so as to form practical guidance and suggestions for urban construction in Xi’an. In terms of the mechanism analysis, this study explored the differences in the residents’ perceptions and the corresponding influencing factors that were relied upon in the statistical methods. The interpretation of the internal mechanism of the relationship between landscape patterns and thermal environment needs to be strengthened. Whether the relevant conclusions could be applied in other regions needs to be verified by more case studies in the future.

## 5. Conclusion

In terms of aiming at the construction of sustainable cities against the background of human health risk prevention, this study investigated the influencing factors of residents’ health risk perceptions of hot days as well as their cognition regarding the cooling effect of urban green spaces, the relationship between health risk perception and residents’ needs of urban ecological construction was also explored. The results showed that most of the respondents believed that the hot days in Xi’an had an impact on them. Housing types, occupation, cooling equipment at work, and outdoor working hours were the significant factors affecting the perception of the intensity of hot days. Specifically, the outdoor workers, residents with relatively poor living environments and with no cooling equipment at work felt more threatened by hot days. Further, gender was found to be a significant influence factor both on health threat and heat-related physical discomfort. Women were more likely to suffer health threats and discomfort symptoms, and usually show a higher vulnerability under the urban thermal risk than men. In addition, the longer the respondents worked outdoors during the daytime in summer, the higher their perceived health risks from heat exposure.

Furthermore, 73.23% of the respondents believed that the cooling effect of urban ecological landscapes in Xi’an has been strengthened in recent years. Monthly income, residential districts, and outdoor working hours were significant influencing factors for their judgment. Workers with longer outdoor exposure were more inclined to believe that the cooling effect of parks was improving. Moreover, relatively more respondents thought the cooling effect of urban green spaces was better than that of water bodies. In terms of landscape patterns, residents who have lived in Xi’an for a longer period tended to agree that the area and shape would affect landscapes’ cooling effect. Respondents showed high acceptance regarding the restrictive effect of landscapes’ area on the cooling effect, most of them were highly educated and had a variety of cooling equipment at work.

Residents’ health risk perception of hot days showed significant impact on their needs of the urban ecological construction. Compared with the respondents who’s mental or physical health were barely threatened by hot days or without any discomfort, the needs of urban ecological construction of whom experienced great health threats or with various symptoms was 4.39 times and 3.51 times, respectively. At the same time, respondents with higher cognition on the constraining effect of landscape pattern on its cooling effect and more optimistic about the cooling trend were also more inclined to strongly support the construction of green cities. However, this study showed that residents in Xi’an City still lacked awareness regarding the mitigation effect of urban thermal environments from the perspective of landscape ecology. Therefore, the following work need to be strengthened in the future. First, to improve the accuracy of the early warnings of hot days, targeted education on health risks and protection from hot days should be popularized for older adult, women, and outdoor workers. Second, to improve residents’ attention and cognition on the cooling effect of urban parks through multiple media, to guide them to take effective travel modes and cooling measures in the face of heat risk. Third, based on residents’ urgent needs for establishing green cities, the close com bination of landscape ecology and urban planning should be further enhanced to promote the construction of a more sustainable and livable city in Xi’an.

## Data availability statement

The original contributions presented in the study are included in the article/Supplementary material, further inquiries can be directed to the corresponding author.

## Author contributions

TZ and RH contributed to conception and design of the study. MY and XM organized the database. RH, GL, and XW performed the statistical analysis. TZ wrote the first draft of the manuscript. MY, GL, XW, and QH wrote sections of the manuscript. All authors contributed to the article and approved the submitted version.

## Funding

This research was funded by the National Natural Science Foundation of China, grant number: 42001097, the Shaanxi Province Association for Science and Technology Youth Talent Support Program, grant number: 20230710, and the College Students’ Innovative Entrepreneurial Training Plan Program, grant number: S202110718094.

## Conflict of interest

The authors declare that the research was conducted in the absence of any commercial or financial relationships that could be construed as a potential conflict of interest.

## Publisher’s note

All claims expressed in this article are solely those of the authors and do not necessarily represent those of their affiliated organizations, or those of the publisher, the editors and the reviewers. Any product that may be evaluated in this article, or claim that may be made by its manufacturer, is not guaranteed or endorsed by the publisher.
