# Supplementary material for: Perceptions of the health risk from hot days and the cooling effect of urban green spaces: a case study in Xi’an, China
Source: Front Public Health. 2023 Aug 22;11:1211164. doi: 10.3389/fpubh.2023.1211164 (PMC10477602; doi:10.3389/fpubh.2023.1211164)
Supplement: Supplementary file 1 [file Table_1.DOCX]

Supplementary Material

Perceptions of the health risk from hot days and the cooling effect of urban green spaces: a case study in Xi’an, China

Tian Zhang^1*^, Rong Huang^2^, Mei Yang^2^, Guohua Lin^2^, Xiaoyan Ma^2^, Xuan Wang^2^, Qian Huang^2^

*** Correspondence:** Tian Zhang: 2019147@snnu.edu.cn

# Questionnaire of urban residents’ health risk perceptions of hot days and the cooling effect of urban green spaces and water landscape

Dear residents:

Hello! We are students from Shaanxi Normal University, we are studying on the cooling effect of the ecological landscape on hot days in the central area of Xi’an City, we look forward to provide suggestions for the prevention of thermal environmental risks in Xi’an City through our study. Your answer will provide a valuable data basis for our study. Sincerely thanks for your cooperation and support.

Instructions:

1. The survey is anonymous, and all the data would be strictly confidential and only used for research, which would not cause any inconvenience to you;

2. Please read the questions and options carefully, choose the answer that best matches your actual situation, please check the options or fill in directly;

3. Only one answer should be selected for all the questions in the questionnaire, except for the questions marked with “multiple choice”.

Date: ______Year ______Month ______Day Name of investigator: ____________

Investigation site: _______District _______Street (town) _____ Community (village)______

# Part 1: Basic Information

| **A1. Gender** | 1. Male 2. Female |
| --- | --- |
| **A2. Age** |  |
| **A3. Physical condition** | 1.Healthy 2. Well 3. General 4. Have disease: ________ |
| **A4. Education** | 1.Junior high school and below 2.High school (secondary specialized school or vocational-technical college)  3.Junior college 4.Undergraduate 5.Postgraduate and above |
| **A5. Occupation** | 1.Public institutions 2.Company employee  3.Service industry 4.Student 5.Outdoor worker 6.Retiree 7.Individual business 8.Unemployed 9.Others: ______ |
| **A6. Monthly income** | 1.Under 2000 CNY 2.2000~4000 CNY 3.4000~6000 CNY  4.6000~8000 CNY 5.Over 8000 CNY |
| **A7. Residence time in Xi’an** | 1.Local people 2. Within six months 3. Six months to two years 4.Three to five years 5. Five to ten years 6.More than ten years |

# Part 2: Daily life

**B1. What is your housing type?**

1.Non-top floor of apartment building 2.Top floor of apartment building 3.Flat building

4.Villa 5.Others: _________

**B2. What kind of cooling equipment do you have in your home (multiple choices)?**

1.Fan 2. Air conditioner 3. Refrigerator 4. None

**B3. Which of the following environments do you mostly work in?**

1. Outside in the sun 2. Outdoor shade

3. Indoor without air conditioner 4. Indoor with air conditioner

**B4. During hot days, what cooling equipment do you use at work (multiple choices)?**

1.Fan 2. Air conditioner 3. Refrigerator 4. None

**B5. How long do you normally need to work outside in a day (single choice)?**

1. Within 1 hour 2. 1~2 hours 3. 2~4 hours 4. 4~6 hours 5. 6~8 hours 6. More than 8 hours

**B6. How do you usually go to work or go out during the summer (multiple choice)?**

1.Walk 2. Bicycle/Electric bicycle 3.Bus 4. Subway

5. Private car 6.Taxi 7.Others: _______

# Part 3: Health risk perception of hot days

**C1. What degree do you think is the hot days? Above_______°C**

**C2. How long do you think the hot days in Xi’an usually lasts?**

During (Month) to (Month)

**C3. How do you feel the hot days affects your daily life?**

1. Barely 2. Minor 3. General 4. Large 5. Great

**C4. What kind of impact will hot days bring to you (multiple choice)?**

1. Affect mood (psychological irritability, anxiety) 2. Travel inconvenience

3. Decrease in travel activities 4. Cost of living increases 5. Decrease in study/work efficiency

6. Cause physical discomfort 7. No influence

**C5. How do you feel the health threat of hot days?**

1. Barely 2. Minor 3. General 4. Large 5. Great

**C6. Have you ever gone to the hospital for medical treatment due to hot days (multiple choices)?**

1. Respiratory discomfort 2. Sleep disorders 3. Cardiovascular and cerebrovascular complications 4. Digestive system diseases 5. Sunburn 6. Sunstroke 7. Others: _______ 8. None

# Part 4: Perception of the cooling effect of urban green spaces and water

**D1. In recent years, do you think the cooling effect of ecological landscape is gradually improved with the construction of urban green spaces and water in Xi’an City?**

1. The cooling effect is getting better 2. Not sure 3. The cooling effect is getting worse

**D2. Do you think the area of water or urban green spaces will have an impact on its cooling effect?**

1. Yes 2. Not sure 3. No

**D3. Do you think the shape of water or urban green spaces will have an impact on its cooling effect? (If not, skip question D5)**

1. Yes 2. Not sure 3. No

**D4. Which shape of water or urban green spaces do you think has the best cooling effect? (A score of 0 is the worst and a score of 5 is the best)**

Water or greenspace landscape in a shape of roundness: _______score

Water or greenspace landscape in a shape of rectangle: _______score

Water or greenspace landscape in a shape of foursquare: _______score

Water or greenspace landscape in irregular shape: _______score

**D5. Compared with urban green spaces and water, which one do you think has a better cooling effect?**

1. Urban green spaces 2. Not sure 3. Water landscape

# Part 5: Personal needs and suggestions

**E1. What policies and measures do you want the government to take to deal with hot days (multiple choices)?**

1. Guarantee water supply and power supply in hot weather

2. Provide high temperature subsidies to needy families

3. Provide heatstroke prevention medicines

4. Increase open spaces with air conditioner

5. Increase green infrastructure

6. Replenish border trees

7. Announce high temperature warnings in time

8. Adjust summer working hours

9. Others:_______

**E2. Facing the hot days, do you think it is necessary to build urban greenspace and water landscape?**

1. No need at all 2. Not quite necessary 3. General 4. Necessary 5. Very necessary

**E3. Do you have any suggestions for the construction of water and greenspaces around your living and working environment?**
